# Identifying strategies to improve access to credible and relevant information for public health professionals: a qualitative study

**DOI:** 10.1186/1471-2458-6-89

**Published:** 2006-04-05

**Authors:** Nancy R LaPelle, Roger Luckmann, E Hatheway Simpson, Elaine R Martin

**Affiliations:** 1 Preventive and Behavioral Medicine, University of Massachusetts Medical School, Worcester, MA, USA; 2 Department of Family Medicine and Community Health, University of Massachusetts Medical School, Worcester, MA, USA; 3 Lamar Soutter Library, University of Massachusetts Medical School, Worcester, MA, USA

## Abstract

**Background:**

Movement towards evidence-based practices in many fields suggests that public health (PH) challenges may be better addressed if credible information about health risks and effective PH practices is readily available. However, research has shown that many PH information needs are unmet. In addition to reviewing relevant literature, this study performed a comprehensive review of existing information resources and collected data from two representative PH groups, focusing on identifying current practices, expressed information needs, and ideal systems for information access.

**Methods:**

Nineteen individual interviews were conducted among employees of two domains in a state health department – communicable disease control and community health promotion. Subsequent focus groups gathered additional data on preferences for methods of information access and delivery as well as information format and content. Qualitative methods were used to identify themes in the interview and focus group transcripts.

**Results:**

Informants expressed similar needs for improved information access including single portal access with a good search engine; automatic notification regarding newly available information; access to best practice information in many areas of interest that extend beyond biomedical subject matter; improved access to grey literature as well as to more systematic reviews, summaries, and full-text articles; better methods for indexing, filtering, and searching for information; and effective ways to archive information accessed. Informants expressed a preference for improving systems with which they were already familiar such as PubMed and listservs rather than introducing new systems of information organization and delivery. A hypothetical ideal model for information organization and delivery was developed based on informants' stated information needs and preferred means of delivery. Features of the model were endorsed by the subjects who reviewed it.

**Conclusion:**

Many critical information needs of PH practitioners are not being met efficiently or at all. We propose a dual strategy of: 1) promoting incremental improvements in existing information delivery systems based on the expressed preferences of the PH users of the systems and 2) the concurrent development and rigorous evaluation of new models of information organization and delivery that draw on successful resources already operating to deliver information to clinical medical practitioners.

## Background

The need for improved access to high quality public health (PH) information has been echoed in various forums involving public health professionals, librarians, and information professionals since the mid 1990s [1-5]. The information needs of the PH workforce have become all the more urgent with the increasing frequency of emergence of new infectious diseases such as severe acute respiratory syndrome (SARS) and Asian bird flu, as well as the increasing concern about acts of bioterrorism, such as spreading anthrax spores via the US Postal Service in 2001.

A major difficulty in meeting these needs is the great breadth of the PH discipline which makes it difficult to identify and collect a body of evidence-based literature to address the growing multitude of specific PH information needs. The PH workforce may be more diverse than any other group of health professionals [6] and includes professionals trained in dozens of disciplines [3,5], ranging from environmental health to veterinary medicine, from sanitary engineering to epidemiology.

Studies of the information needs of PH professionals have addressed information seeking behaviors of PH workers, obstacles to information access, and defining and classifying the specific types of information needed. Reports of studies focusing on how PH workers look for the information they need note that many PH professionals have been slow to adopt electronic information-seeking behaviors, sometimes because of the time required for users to acquire the requisite skills and other times because access to electronic databases is not available [7-9]. Studies focusing on obstacles to electronic access to and use of evidence-based information in the PH field [1-3,5,10-13] have identified many relevant obstacles including: (1) limited awareness of the importance of evidence-based information to inform practice and lack of encouragement from opinion leaders to seek it; (2) limited awareness of what information is available electronically and from what sources; (3) limited access to computers, the Internet or email; (4) limited skills needed to access the information sources and lack of ease of use; (5) a diverse array of content needs requiring access to databases from many disciplines (and in other languages); (6) limited time to sift through the poorly filtered information that is returned by searches using sets of search terms inadequate for PH concerns; (7) limited ability of decision-makers to appraise the methodological quality of research; and (8) the paucity of systematic reviews of PH topics.

Lynch [14] suggested that information accessing needs among PH professionals often focus on immediate problem-solving and not on answering open-ended academic research questions. Investigators focusing on the kinds of information needed by PH professionals [5,6,14] have noted the need for diverse kinds of information including the grey literature and unpublished studies, practice guidelines, research studies and systematic reviews. These investigators also point out the need for information that is effectively summarized and synthesized. There is also a need for linkages among multiple databases as well as providing access to databases related to best practices, outcome measures, statistical information, policy updates, and information that may be unique to a particular location or region. Nutbeam [4] has formalized a valuable four-level typology of increasingly informative levels of research knowledge in the PH field. Nutbeam's model does not, however, include grey literature that may be needed to assist PH decision makers when the evidence needed to inform urgent PH issues is incomplete.

Access to evidence-based public health information has become a growing concern for medical librarians. In 1997, the National Library of Medicine along with the Centers for Disease Control and Prevention (CDC) and other agencies launched the Partners in Information Access for the Public Health Workforce project to begin to address this concern. The Evidence-Based Practice for Public Health (EBPPH) Project at the Lamar Soutter Library of the University of Massachusetts Medical School was initiated in 2001. This Project initially focused on identifying resources available to clinical medical practitioners and PH practitioners for locating, summarizing, synthesizing and disseminating evidence-based information. It then compared resources available to clinical medical practitioners to those available to PH practitioners. We found that there were many more types of resources focused on clinical medical practice than on PH practice. The clinical medical resources were based on several different models of information search, summary, synthesis and delivery, and some of the most promising models had little or no presence in the PH arena.

To explore and address this gap, the EBPPH project sought to examine and classify the features of the clinical evidence-based medicine resources, to assess their potential for improving access to the PH literature, and to develop new models that could effectively address the unique needs of PH professionals. EBPPH also works on identifying other existing projects aimed at synthesizing, summarizing or improving access to evidence-based PH information and publishes links to effective evidence-based resources it has identified via its website [15].

This article presents the results from a qualitative study undertaken by the EBPPH project that combined three objectives that previous investigators had generally pursued individually: (1) characterization of information needs of PH practitioners, (2) identification of typical information seeking behaviors, and (3) assessment of barriers to information access. We have used the insights gained from the study to inform the construction of an extended classification of the types of information needed by PH professionals and of a hypothetical model for PH information access that could meet their needs for access to diverse credible sources.

## Methods

### Operational definitions

Jenicek [16] defines evidence-based public health practice as the conscientious, explicit, and judicious use of current best evidence in making decisions about the care of communities and populations in the domain of health protection, disease prevention, health promotion, and health maintenance and improvement. We too are using the term "evidence-based" to refer to the best available evidence that is both credible and of the highest known quality.

### Research process overview

The research project team of four consisted of 1) the Lamar Soutter Library Director with a DA degree, 2) one of her staff members with an MPH degree (both had formal training in qualitative research techniques provided by the qualitative research consultant), 3) a medical consultant with an MD and expertise in medical informatics (quantitatively trained with prior experience working with the qualitative research consultant on other qualitative studies), and 4) a qualitative research consultant with a background in software development, a PhD in organizational and human development, and expertise in teaching and applying qualitative methods in a variety of studies related to medical and PH interventions. All four participated in the development of interview questions and the conduct of focus groups as well as reviewing the findings and developing the models.

**Figure 1:**
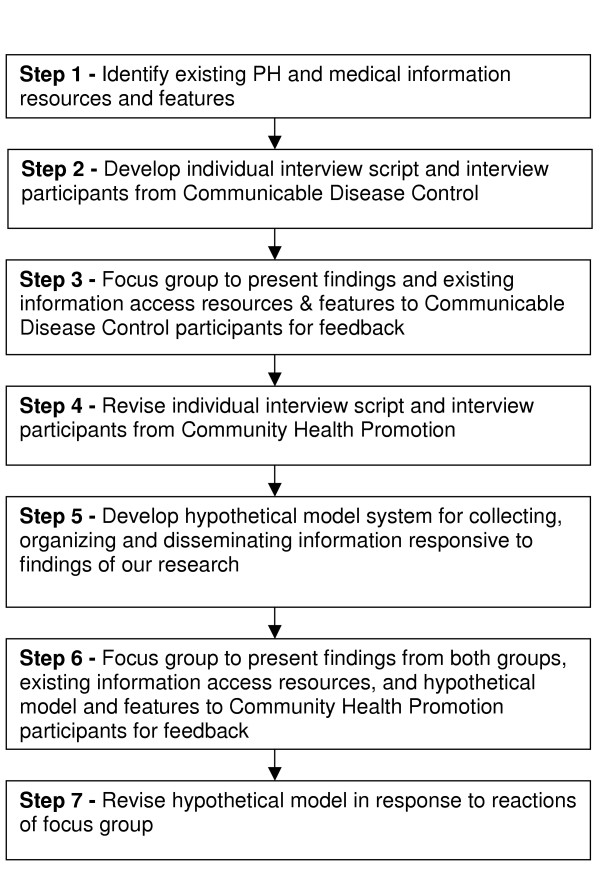
EBPPH research process steps.

The EBPPH research process was a seven-step process (See Figure 1). The first step was to identify and characterize existing information resources available to medical and PH professionals. The second step was to collect data on information accessing needs, behaviors, and barriers via individual interviews from one domain of PH practice, communicable disease control. Thirdly, after findings were analyzed, they were presented to a focus group of PH professionals previously interviewed along with examples of information accessing resources currently available to gather additional input on information accessing needs. In the fourth process step we used information gathered to revise the individual interview script and make it more specific for interviewees in a second, but related domain of PH practice – community health promotion. The fifth step involved analysis of this new data and construction of a preliminary hypothetical model with features to meet the information accessing needs identified by the participants. In step 6 an analysis of the findings from both domains, examples of existing resources, and the hypothetical model were reviewed in a focus group with Community Health Promotion staff previously interviewed. As a result of step 6, we revised the hypothetical model to reflect the feedback gathered in the last focus group.

The research protocol used for this study was approved by the Committee for the Protection of Human Subjects in Research at the University of Massachusetts Medical School, IRB Docket #H-10507.

### Recruiting process and sample selection

We chose subjects for our study from the Massachusetts Department of Public Health (MDPH) because of ongoing relationships between investigators and MDPH employees and because of the proximity of the Department offices to the EBPPH project site. We focused on the Bureau of Communicable Disease Control (BCDC) and the Bureau of Family and Community Services' Community Health Promotion (CHP) groups because they are both relatively large groups and because these two groups were likely to represent a significant portion of the broad spectrum of information needs in PH in general. One group deals primarily with prevention and control of communicable diseases (BCDC) and the other with health promotion related to prevention and control of chronic disease (CHP).

The Director of each sample work group was asked to provide names of individuals in his/her group who most often needed access to information from multiple sources. Twelve potential interviewees were selected by the BCDC Director from the program areas of tuberculosis prevention and control; epidemiology and immunization; sexually transmitted disease (STD) prevention and control; refugee and immigrant health; and library services. Eight potential interviewees from the CHP group were selected by their Director in the areas of cancer, diabetes, and cardiovascular disease prevention and control; nutrition and physical activity; and women's, men's and elder health. An email message was sent to each of those selected requesting agreement to participate and interviews were scheduled by either email or telephone. All twelve of the BCDC interviewees suggested participated in the individual interviews while seven of the eight approached from CHP participated. Seven of the BCDC informants (60%) participated in a follow-on focus group at a later time. Three CHP employees previously interviewed (43%) participated in the CHP follow-on focus group. The professionals in our sample ranged from information technologists, librarians, trainers and program directors to medical directors and division directors. Informants held various degrees in nursing, public health, medicine, veterinary medicine, and other specialties.

### Data collection

Data were collected from the two sample groups first via 45-minute individual interviews with all participants. During later focus groups, lasting about an hour and a half, preliminary findings were reviewed and validated and additional data were collected as participants reacted to PowerPoint slides presented describing existing information sources and accessing features. A hypothetical model for PH information access was also presented at the second sample focus group for feedback. All individual and group interviews were audiotaped. Individual interviews were transcribed verbatim, and the qualitative research consultant listened to the focus group tapes and took notes of study participants' comments related to information accessing features presented as close to verbatim as possible.

### Analyzing data and validating coding

Based on a technique developed by LaPelle [17], we employed Microsoft Word to transcribe data into tables of text responses where coding and sorting of text segments can be done based on theme codes. Our thematic analysis approach rests heavily on the qualitative research techniques described by Crabtree and Miller [18-20], Miles and Huberman [21], and Patton [22]. Transcribed textual data from interviews were reviewed by the qualitative research consultant through a continuous process of comparing data segments to other data segments, looking for similar or repeated ideas to be conceptualized as themes. A continuously evolving codebook was developed defining themes identified in the interview script as well as subthemes as they emerged from repeated readings of the data. The transcripts in the Microsoft Word table structures were coded thematically in a table column adjacent to the relevant text segment using the preliminary draft of the codebook. Codebook development and coding were conducted solely by the qualitative research consultant. After sorting coded text segments via the Microsoft Word Table Sort function, coding validity was assured by reviewing the text that sorted into each code, correcting those that were miscoded and resorting. Some themes were further deconstructed creating additional subthemes after sorting when this seemed appropriate. Within-transcript analyses and data reduction were done, proceeding to cross-transcript analyses. A comparison table was constructed to compare summarized responses related to each significant theme across participants for each group. Subsequently findings were compared across groups. At each stage in the data analysis and reduction, findings were reviewed with other members of the project team. Additionally, findings were reviewed with participants from each sample at the focus groups for further validation.

### Step 1 – identification of existing information access resources and features

The EBBPH medical consultant identified existing resources delivering information to medical practitioners and to PH professionals. These were advertised via mailings, email promotions, and vendor displays at academic meetings and identified in discussions with clinical and PH professionals who were intensive consumers of medical and PH information. He also performed informal Internet searches using Google. Through the use of trial and full subscriptions, the consultant informally explored the resources he identified and catalogued the features of these resources. The key access, formatting and content features these resources offered are listed in Table 1: (1) keywords for searching large collections of research reports and reviews, (2) pre-formulated search filters, (3) automatic notification (e.g. periodic emails to subscribers with information on recently published research reports), (4) abstracts or summaries of research reports sometimes associated with commentaries, (5) systematic reviews, (6) evidence-based guidelines, (7) comprehensive knowledge sources, (8) within article indexing with links that allows access to the specific information needed, and (9) archiving capabilities.

### Steps 2 and 3 – individual interview and focus group data collection for BCDC

The individual interview script was developed by the project team. The BCDC script included questions about the types of work tasks informants performed that required access to information, currently used and preferred information sources, preferred format for research information, current barriers to information access, and desired enhancements for access [see Additional file 1]. Individual interviews with BCDC professionals were done in person at their workplace by the project's qualitative research consultant hoping to observe their electronic information accessing behavior where possible. Those who were very computer literate or had sizeable offices met with her in their offices and were comfortable demonstrating how they accessed their preferred electronic sources. However, many either had no space in their offices to hold the interview or accessed most of their information in ways that were not easy for us to observe. The individual interviews with BCDC informants took place in the fall of 2003.

A follow-on focus group was held in the spring of 2004. The focus group agenda for BCDC included reviewing a summary of findings from their interviews for validation followed by a PowerPoint presentation on several existing website examples of PH information organization and dissemination (see Table 1) to ascertain their familiarity and preference for specific features of these models. The focus group was conducted by project team members without a detailed script.

### Step 4 – initial data collection for CHP

The individual interview script for CHP participants used the BCDC script as a basis but was modified by the project team to add more specific questions regarding listservs providing links to current information, systematic reviews and comprehensive knowledge bases based on inconclusive findings in these areas from interviews with the BCDC participants [see Additional file 2]. Individual interviews with CHP professionals were conducted by the project's qualitative research consultant by telephone since most in-person interviews with BCDC participants had not yielded opportunities to observe their information accessing behavior directly. The individual interviews with CHP informants took place in the summer and fall of 2004.

### Step 5 – hypothetical model development

Following the individual interviews with CHP informants, we developed a preliminary hypothetical model of information reformulation, organization and access based on our interpretation of CHP informants' responses to the interview questions and prior findings from the BCDC informants.

### Step 6 – focus group with CHP participants

The focus group agenda for CHP included reviewing a comparison of findings from BCDC and CHP, validation of CHP findings and a review of existing information accessing website examples (see Table 1), followed by specific questions [see Additional file 3] about desirability of specific resource features. Additionally the hypothetical model that addressed most of the articulated needs was presented and additional questions were asked about the use of PubMed [23] as a foundation for implementing the model and how PubMed would need to be enhanced to evolve into an implementation of the hypothetical model. The focus group was conducted in the early spring of 2005 by project team members.

## Results

### Work context

Professionals at MDPH have a wide variety of needs for information and varied significantly in their level of skill in accessing it. All MDPH staff we interviewed had desktop computers and access to the Internet. MDPH itself hosts a multi-faceted website that provides access both for employees and for the general public to programmatic information, statistical databases and MDPH documents. The main MDPH offices are located in multiple sites in the Boston area. The CHP group and the BCDC are located in different office buildings, each of which includes a small library staffed by part-time librarians offering document search and retrieval services. However, the urgency of information needs differed widely within and across programs, and use of external electronic information sources also differed widely due to variation in both need and skills.

### Tasks requiring external information access

The nature of the work of the two groups also differs. BCDC is concerned with both established and emerging communicable diseases and needs fast-breaking news about emerging diseases such as SARS as well as evidence-based information about more established diseases like tuberculosis. CHP deals primarily with health promotion related to chronic diseases and is involved in working with external coalitions to develop statewide collaborative prevention and control plans for diseases such as cancer, cardiovascular disease and diabetes. However, there is also significant overlap in the information accessing needs of these two groups.

### Information needs and information-seeking behaviors

Six distinct categories of information emerged from the participants' statements about the kinds of information they required in their work: (1) early reports on newly identified health risks and preventive behaviors; (2) early reports on emerging practices and programs, usually descriptive in nature; (3) information on evaluated new interventions known to be effective; (4) syntheses of knowledge on established public health threats and practices as typically found in reference texts; (5) published research reports, including meta-analyses and systematic reviews as found in peer-reviewed journals, often based on formal research designs; and (6) evidence-based guidelines. [See Additional file 4 for informant quotes supporting these 6 categories of information needs.]

Examples of the kind of information currently accessed by the participants in each of the six categories and typical associated sources of information can be found in Table 2. Those we interviewed in their offices demonstrated how they accessed many of the source examples in Table 2, provided website information, or gave us sample copies of information available from these sources. The sources included websites, electronic automatic notifications often via listservs, journals, presentations at meetings, and personal communications. Websites and general search engines were commonly used to seek out the most relevant information. Email was a key means for receiving automatic notification via listservs and exchanging a variety of types of information. Existing listservs for PH professionals typically provide information from news sources and selected titles, abstracts, and links to recently published journal articles and other documents relevant to specific disciplines or diseases. A few informants who were less comfortable with Internet access to information still relied on hard copies of relevant journals that were circulated within their workgroup. The telephone and attendance at conferences provided other important means of accessing information, especially on emerging health threats and new PH practices.

### Limitations of existing mechanisms of information access

We found that there were significant limitations on the available means of information access identified by participants that could be met by improving electronic access mechanisms. Stating that there were too many relevant websites to search them all effectively or regularly, both groups wanted one portal access to all categories of information via a good search engine. To address delays in becoming aware of important new information, they also wanted automatic notification of newly available information in areas specific to individual interests. They felt websites and automatic notification systems such as listservs were complimentary. Participants in both groups reported feeling bombarded with unfiltered, often duplicative information in emails and from participation in listservs with no way to screen out irrelevant information. They reported similar difficulties dealing with irrelevant and duplicative returns from searching websites because PH-specific keywords are not standardized or used effectively by search engines. Both groups expressed a need for better mechanisms for selecting and filtering information sought from listservs and via search engines.

Both groups noted limitations on access to information in selected PH sub-domains of interest. BCDC professionals noted information gaps in the areas of STDs and refugee and immigrant health. CHP professionals cited limitations on information in areas such as environmental links to cancer, elder health, legislative and policy change nationally and in other states, and newly identified health risks and healthy behaviors. CHP and BCDC informants identified problems in accessing relevant information from related domains outside of traditional PH domains such as in the literatures of marketing, human resources management, organizational behavior, operations management, and others. They would like to be able to formulate searches, for example, that could access a broad range of databases to find evidence related to: (1) return on investment for worksites implementing health programs; (2) programs that have been developed in worksites related to communicable or chronic disease prevention; (3) effective educational strategies to reach employees in worksites; (4) effective interventions to motivate, men, women, elders and other specific population groups to take care of their health; (5) effective quality improvement projects in healthcare organizations; and (6) best practices related to communicable diseases in emergency rooms. They also identified limitations on access to grey literature as well as systematic reviews and full-text of journal articles. Informants stated that not many extensive sources exist for systematic reviews and summary information of interest to PH; however, a few participants were not aware of those that do exist, e.g., the Guide to Community Preventive Services [24].

Many also expressed difficulty keeping track of information they wanted to save for future access and wanted better mechanisms to archive information accessed earlier in a way that could facilitate easy retrieval. Informants also expressed needs for training in electronic accessing skills and the availability of human-mediated searching via article retrieval services.

### Hypothetical model

**Figure 2:**
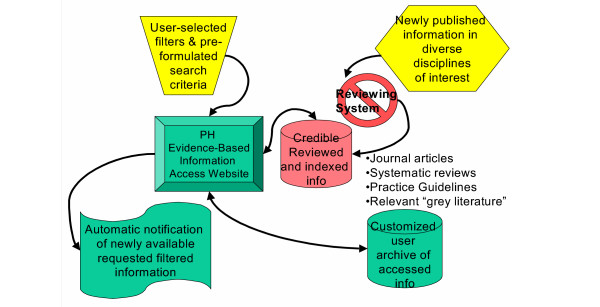
Hypothetical model for PH information access presented to CHP informants.

In response to the information content, format and access concerns raised by informants we developed a hypothetical model for PH information access grounded in findings from our data analysis [See Additional file 5 for quotes supporting the features included in the model.] The model (See figure 2) includes PH-specific keywords, user-selected filters, and pre-formulated search criteria for tailoring information sought via both automatic notification systems and search engines; an automatic notification mechanism that would send information to users that has been filtered according to the user's filtering requests; a scanning and reviewing system to locate, critique/review, and provide links to newly published information relevant to PH professionals, and a customized archiving database of credible information accessed by system users.

In early 2005 when we presented this model to the CHP participants, the component at the top right said simply "newly published PH information." We initially presented the model as an approach to accessing and delivering information that appeared in PubMed. Respondents stated that to be very useful the model would have to facilitate access to information from other sources and disciplines not represented in PubMed. It should also reference descriptions of best PH practices and other information often found in the grey literature. In response to this concern, we added the phrase "in diverse disciplines of interest" to the model component on sources of information in the upper right of figure 2. Respondents liked the idea that information would be reviewed before it was disseminated. They expressed a desire to receive a narrative critique of the information from the reviewers and not just numerical ratings expressing the reviewers' findings.

Other information accessing needs identified fell primarily into the category of grey literature and statistical data. These included the ability to search conference websites via a single portal for cutting edge information and expert contact information; access via keyword search to information the CDC has on programs being implemented in other states; easy access to news articles about what is happening statewide related to health programs in schools and other venues; and a database "mecca" for surveillance data.

## Discussion

In general, our respondents confirmed many of the findings of others who have investigated PH information needs [5,6,14] including the need for access to a wide variety of types of information from a number of different disciplines and the need for improved means to effectively and efficiently identify the information most relevant to specific problems. We believe that our study also adds detail, depth, and updating to preexisting characterizations of PH information needs in several areas: (1) categories of information needed, (2) typical present-day sources of information and common means of accessing information, and (3) perceived limitations of access and proposals for overcoming those limitations.

### Categories of information

**Figure 3:**
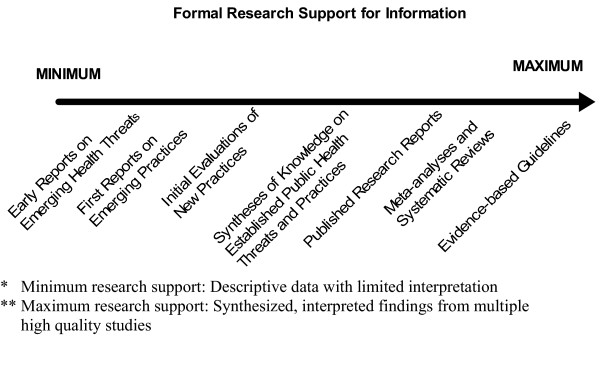
Continuum of research support for common categories of public health information.

Six categories of PH information emerged from both groups in our study (see Table 2). The six categories can be arrayed along a continuum based on the amount of available research evidence supporting each of the categories. On the continuum shown in figure 3, we have separated category 5 into two categories: published research reports and meta-analyses/systematic reviews to emphasize the difference between these two resources. The resulting seven-category continuum ranges from limited research support for early descriptions of emerging health threats and new interventions on the lower end of the continuum, through increasing amounts and quality of evidence for the types of information farther along the continuum.

Our classification of PH information extends the typology developed by Nutbeam [4] in that it covers the full range of information needed to inform PH decision-making from basic descriptive data to evidence-based guidelines. Our categories 3–7 are also an expansion of what Brownson [25] calls type 2 evidence or evidence that focuses on the relative effectiveness of specific interventions to address a particular health condition.

Like clinicians practicing medicine, PH practitioners are regularly confronted with new problems and complex tasks that require a timely response, often before researchers can complete studies to offer guidance. Our study confirms the findings of others that PH practitioners need access to information at all levels of research support on the continuum in figure 3. Anderson et al [26] highlighted the need for PH practitioners to access information supported by limited evidence and to weigh the credibility of this information before using it in PH decision-making related to urgent issues that cannot wait for conclusive evidence to accumulate.

### Information access

Participants identified several limitations in information access and in some cases suggested means for overcoming those limitations. The limitations could be classified in three broad categories: (1) limitations affecting timely and convenient delivery of information including limited access to electronic full text of journals, (2) limitations in access to all categories of information in some content areas (i.e., disciplines outside traditional PH) and in the grey literature, and (3) limitations in locating information that is available, including problems with search terms and in organizing archives.

Participants did not specifically identify as a problem the very limited availability of summaries, commentaries and critiques of PH studies, even after we had presented to them examples of these resources from both PH and clinical medicine. We hypothesize that one reason informants did not report this limitation is that they have had little or no experience working with these types of resources. It is possible that as more summaries, commentaries and critiques become available to PH practitioners that their desire for more of these resources may increase. Participants did identify the need for access to systematic reviews of interventions that are more population-based and PH-oriented. One of the few resources we identified that has begun addressing this need broadly is the recently developed Health-Evidence.ca website [27] that provides an online registry of systematic reviews of PH interventions. This project, funded by the Canadian Institutes of Health Research and conducted by Dr. Maureen Dobbins, searches for systematic reviews in a variety of databases of published literature including PubMed, CINAHL (Cumulative Index to Nursing and Allied Health Literature) [28], EMBASE [29], Sociological Abstracts [30], BIOSIS [31], and PsycINFO [32]. Selected reviews are rated by two independent reviewers. Review information is stored in a searchable registry. The site also provides summaries of the reviews but has not yet included access to unpublished work. Other databases that present summaries of synthesized evidence-based practice information for public health professionals are more limited, such as the Cochrane Health Promotion and Public Health Field website [33] which addresses only a tiny fraction of common public health issues currently, or more specialized, such as the Australian databases for occupational therapists, OTseeker [34,35], and physiotherapists, PeDro [36,37].

### Overcoming access limitations: barriers and options

The informants offered a number of potential approaches for overcoming the limitations they identified in timely delivery, access, and location of information. They stated a preference for overcoming these limitations via enhancements made to sources and means of information access they were already using such as PubMed and listservs.

To overcome limitations in timely delivery of information, participants suggested that improved access to the full text of journals in electronic form would be critical. Many of the biomedical journals that publish articles relevant to PH professionals are already available in electronic versions, but the cost of offering access to these journals to dozens or hundreds of PH practitioners can be daunting for any local or state public health entity. However, there are some examples in other countries where this has happened. The Clinical Information Access Program (CIAP) [38,39] in New South Wales, Australia, and similar systems in other Australian states, have provided access to over 55,000 clinicians in the public health workforce with access to in excess of 400 such publications by government negotiations for reasonable cost licenses for very large populations. An extensive evaluation of this system is being undertaken to examine how availability of such information influences practices.

Many informants from both groups in this study were already receiving news and automatic notification of newly published studies via listservs. Unfortunately comprehensive listservs are available for only a few PH disciplines (e.g., TB Update [60]). Our findings suggest that developing listservs that organize and deliver relevant new information for more PH disciplines may be an effective way to meet some key information needs.

A serious limitation of listservs identified by several subjects is that they tend to deliver large amounts of information in formats that make it difficult for a user to quickly seek out specific information relevant to his/her interests. We are aware of several technical means that some listserv administrators are using to deal with this problem. Items in an email can be linked to an index that appears as the first entry in the email. Links take the user to content contained in the email or on a website. Content developers for clinical medical automatic notification services are using two other techniques to help users avoid receiving irrelevant content: (1) breaking content into many subdomains and allowing users to select to receive information from specified subdomains and (2) engaging volunteer users in a process of filtering content by having them rate the quality and relevance of new information before it is delivered to all the users. Adoption of some or all of these strategies by the developers of PH listservs could effectively address some of the concerns raised in our study.

The request for access to a broader base of information from fields outside of traditional PH disciplines was driven by the need to develop effective evidence-based policies in emerging areas of PH practice such as designing environments to promote physical activity or increase pedestrian safety and developing economic justifications to encourage businesses to introduce health-related programs. CHP informants suggested that access to information from other industries, professions and disciplines would be needed including information from the realms of social marketing, advertising, sociology, engineering, human resources management, and others. Some of this information is contained in journals serving the disciplines of interest, which can be searched through reference databases such as CINAHL, EMBASE, Sociological Abstracts, BIOSIS, PsycINFO, and others. Access to information in these journals by PH professionals would require establishing access to the reference databases and to print and/or electronic access to the journals themselves. In domains beyond the biomedical, it is likely many PH professionals, like those in this study, do not have skills to efficiently gather evidence or formulate searches. Either the appropriate keywords and data sources are not known to PH professionals, or concepts and keywords that are familiar to them, such as "intervention venues" or the "built environment" related to injury prevention, are not used in the other disciplines.

Even when PH professionals have ready access to a database of relevant biomedical information such as PubMed, our subjects indicated that locating useful information is hindered by the paucity of PH-oriented search terms. For example, they stated a need for search terms like "evaluated", "cost-effective", and/or "population-based interventions". Development and adoption of more standardized PH search terms could make searches more effective and also facilitate the task of filtering listserv emails. Developers of the Health-Evidence.ca website have not only provided access to systematic reviews of PH interventions but have also made strides in addressing the need for PH-specific search terms. These developers have generated their own set of PH keywords related to domains of PH, population characteristics, intervention sites, intervention strategies and types of reviews (meta-analysis, narrative or systematic).

Pre-formulated search filters use combinations of existing search terms to facilitate identification of information that meet predefined criteria. In clinical medicine, a set of filters aimed at identifying publications reporting high quality, evidence-based studies has been developed, tested and made accessible for routine use in PubMed (PubMed Clinical Queries [41]). The Partners for Information Access for the Public Health Workforce project [42] has developed dozens of pre-formulated searches aimed at locating information in PubMed relevant to specific Healthy People 2010 goals [43]. Wilczynski et al [44] have developed a set of search filters for PubMed to enable efficient retrieval of articles relevant to healthcare quality and costs based on several criteria related to methodological rigor (appropriateness, process assessment, outcomes assessment, costs, economics, and qualitative research) [45]. Although some of our respondents were familiar with some available filters, they did not appear to be using filters on a regular basis to access information. There is clearly a need to develop and promote more and better search terms and filters for PH since these will be important for locating studies for reviews and for skilled and interested users.

### Limitations

The field of public health is so broad that any study of this sort is limited to collecting data in a few domains in a limited number of settings. Caution is advised in generalizing our findings more broadly to communicable and chronic disease prevention and control practitioners, especially those in city or county health departments or working at the federal level. Also, further investigation is needed in other unrelated PH disciplines to test applicability of findings in these areas because of the wide variation in content and nature of PH practices. Another limitation is that our interviews and group discussions may have been biased towards PH workers with the most interest in information issues.

## Conclusion

It seems clear from our study that it is likely that many critical information needs of PH practitioners are not being met efficiently or at all; however, incremental improvements to PH information access are being made. Projects like Health-Evidence.ca and Partners in Information Access for the Public Health Workforce are emerging. The increasing availability of all types of information on the Internet and ongoing improvements in search methodology and website organization are also occurring, but the task is complex and enormous, and progress seems to be slow. It is our assessment that tools and resources available to clinical medical practitioners are more advanced and sophisticated than those available to PH practitioners. The former include tools such as information sources that summarize and critique findings from research reports (e.g., Cochrane Systematic Reviews) [46]; those for identifying and accessing a broad range of information including basic facts, research findings, expert opinion, and sometimes associated evidence ratings (e.g., electronic texts such as UpToDate) [47]; automatic notification services (e.g., bmj.com Email alerting service) [48]; and periodic, discipline-specific literature updates with summaries and commentaries (e.g., Journal Watch) [49]. We believe one reason for this is that the market for information-related products for medical practitioners is large enough and lucrative enough to attract large investments in many new and creative ventures. The result is a proliferation of well-designed electronic texts, multiple sources of expert summary and critique of the literature, sophisticated and highly tailored automatic notification systems, and many other resources. A second reason that PH practitioners have fewer resources available to them is a lack of good primary research in PH that can be systematically reviewed [3,26]; however, PH practitioners also do not always have ready access to the best evidence that is available such as basic facts and expert opinion.

Based on our findings from this study, we believe that PH could benefit from a dual strategy for advancing information access. One strategy is the promotion of incremental improvements in existing information sources and access mechanisms, such as providing better and more PH-specific search filters for PubMed and developing more sophisticated listserv applications for disseminating automatic notifications. Initially respondents in this study seemed to favor improvements in simple tools and resources they were familiar with over new and more complex models of information access. This suggests that incremental changes may be more rapidly accepted and adopted than new and unfamiliar systems. However, the generally positive reception to our proposed information access model by the CHP informants, suggests that a well-designed new system that clearly addresses expressed needs could be well received. Given that products are emerging that address some of these needs such as Health-Evidence.ca, the Partners in Information Access for the Public Health Workforce website, and the PubMed Health Services Research Queries discussed earlier, there is a clear need to integrate complementary efforts rather than duplicating what has already been done. This cannot be accomplished without collaboration across projects, stakeholders, funding sources, and even national borders. Additionally government entities may be able to negotiate reasonable cost licenses for large PH populations to provide access via emerging systems to many journals relevant to PH practice as has been done in Australia.

We suggest that organizations concerned about PH practitioners' access to information should consider joining forces to sponsor further research to evaluate emerging information systems, fund collaborative research projects, and encourage small scale trials of some new systems for information access such as the model system we propose while at the same time continuing to foster ongoing incremental changes. Those systems that are positively received by practitioners and can show significant objective improvements in the efficiency and effectiveness of information access for a reasonable cost could then be considered for broader dissemination.

## Abbreviations

Agency for Healthcare Research and Quality, AHRQ; Bureau of Communicable Disease Control, BCDC; Centers for Disease Control and Prevention, CDC; Community Health Promotion, CHP; Cumulative Index to Nursing and Allied Health Literature, CINAHL; Evidence-Based Practice for Public Health, EBPPH; Massachusetts Department of Public Health, MDPH; public health, PH; severe acute respiratory syndrome, SARS; sexually transmitted disease, STD

## Competing interests

The author(s) declare that they have no competing interests.

## Authors' contributions

NRL provided qualitative study design and implementation expertise in drafting the preliminary interview scripts, conducting individual and group interviews, analyzing the data, and drafting preliminary manuscript text, tables and figures. RL provided medical and informatics expertise, identified existing clinical medicine Internet-based information resources, reviewed key features of existing information resources with focus group participants, and made substantial contributions to the manuscript. ERM served as principal investigator for the study, assisted in the development of the interview and focus group scripts, provided background information for the manuscript, and participated in the review of the manuscript. EHS identified and classified existing PH information resources and assisted with the development of interview and focus group scripts, with conducting focus groups, and with the coordination of the study and revision of the manuscript.

## Pre-publication history

The pre-publication history for this paper can be accessed here:



## Supplementary Material

Additional File 1 Script for BCDC key informant interviews at DPH. Click here for file

Additional File 2 Script for CHP key informant interviews at DPH. Click here for file

Additional File 3 Some Focus Group questions for the DPH/CHP folks after the powerpoint presentation. Click here for file

Additional File 4 Quotes Supporting Information Need Categories. Table of themes and supporting quotes. Click here for file

Additional File 5 Quotes Supporting Information Accessing Feature Needs and Preferences. Table of themes and supporting quotes. Click here for file

## Figures and Tables

**Table 1:** Information accessing and formatting features and examples of each reviewed with informants

**Information Accessing or Formatting Features**	**Website Examples of Specific Features Reviewed with Informants**
Keywords for searching published literature for articles of interest	PubMed [23]
Pre-formulated search or screening filters	Partners in Information Access for the Public Health Workforce [42] PubMed Clinical Queries [41]
Automatic notification (alert systems and topical listservs)	AJPH Online Subscriber Help & Services[50] SafetyLit [51] TB-UpDate from the CDC [60]
Abstracts, summaries and expert commentaries	Journal Watch [49] Evidence-based Healthcare and Public Health [52]
Systematic reviews	Cochrane Systematic Reviews [46]
Evidence-based guidelines	Guide to Community Preventive Services [24]
Comprehensive knowledge collection	CDC website [53], UpToDate [47]
Within article indexing	UpToDate [47]
Archiving of information sought	SafetyLit [51]

**Table 2:** PH Information Need Categories, Examples of Needs and Related Information Source in Two Community Health Bureaus

**Information Category**	**Communicable Disease Control**		**Community Health Promotion**	
	**Information Need**	**Source Examples**	**Information Need**	**Source Examples**

1. Early reports on newly identified health risks and preventive behaviors	Fast breaking news or alerts re: Asian Bird Flu or West Nile Virus	News media ProMed [54] Epi-X [55]	Emerging risks and newly identified behaviors that reduce chronic disease risk (e.g. physical activity)	News media
2. Early reports on new practices and programs	Reports on SARS interventions	Conferences, informal networks, listservs	Promising prevention programs at work sites	Conferences, informal networks, listservs
3. Evaluations of new interventions	Effective STD interventions	STD Advisor (newsletter)	Effective elder health interventions	Conferences, grey literature
4. Reference Information on health risks and practices	Tuberculosis knowledge synthesis	Medical Reference Books, American thoracic Society [56], CDC [57]	Diabetes knowledge synthesis	Medical Reference Books, American Diabetes Assoc. [58], CDC [59]
5. Published articles and reviews	Research articles on communicable disease prevention and control	PubMed [23], journal-specific search engines, TB Update [60]	Research articles on chronic disease prevention and control	PubMed [23], journal-specific search engines
6. Evidence-based guidelines	vaccine safety	Institute of Medicine [61]	Prevention and control of heart disease	AHRQ (Agency for Healthcare Research and Quality) [62], National Heart, Lung and Blood Institute [63]
