# Supplementary material for: Identifying strategies to improve access to credible and relevant information for public health professionals: a qualitative study
Source: BMC Public Health. 2006 Apr 5;6:89. doi: 10.1186/1471-2458-6-89 (PMC1456961; doi:10.1186/1471-2458-6-89)
Supplement: Additional File 1 — Script for BCDC key informant interviews at DPH. [file 1471-2458-6-89-S1.doc]

# Script for BCDC key informant interviews at DPH

# 10/7/2003

As part of a project funded by the CDC and ATPM (Association of Teachers of Preventive Medicine) and conducted by the UMass Medical School Library, we are conducting research to determine the information access needs of public health professionals. In order to gain some preliminary focus for the project, we will focus first on the area of communicable disease control, and your Director has graciously offered to assist us by providing access to his staff members from whom we will do our initial data collection.

We are interested in the types of information needed by public health professionals to do their work and the most effective information access mechanisms for them. Several of the key informants we have already interviewed from the Bureau recommended that we talk to you as someone who is a resource for providing critical and/or current work-related information to those in the bureau. I would like to start by first asking you a few questions about your responsibilities related to information.

1. What is your job and how does it fit into the overall Bureau?
2. What kinds of questions and information needs come up from those you support in your work that you need to address? (Give examples).
3. For the/one of the examples you just gave, what would be the steps in the process you would use to find the information to address the need starting with the first thing you might do?
4. What kinds of information about communicable disease control do you seek regularly and how do you pass it on to the end users?
5. Where do you currently get the communicable disease-related information you disseminate?
   1. Web sites, web searches, online literature searches, online or hard copy journals, emails from co-workers, email distribution lists, online newsletters, books or other hard copy documents
   2. What information sources are the most credible and or up-to-date
   3. What information format is the most useful to you and those you support? (i.e., indexes, abstracts, original articles, reviews, commentaries, etc.
   4. Any other sources?

For offline information sources identified, continue with questions 10-13.

For online information sources identified, continue with questions 6-9.

1. What are your preferred methods for accessing this kind of information online?
2. What do you like most about this online information access methods?
3. What is the process you use to find the information you need online?
   1. Would you be willing to walk me through a recent example of this?
4. What would make this online information access method even more to your liking?

Go to question 15.

1. What are your preferred methods for obtaining this kind of information?
2. What do you like most about obtaining information this way?
3. What is the process you use to find the information you need in this source?
   1. Would you be willing to walk me through a recent example of this?
4. What would make obtaining information you need from this source even more to your liking?
5. Considering all the sources of information and the information formats used in each, which sources and formats do you prefer, or are most useful to you?
6. What is your reaction to this list of journals and websites we have identified in terms of awareness and usefulness of these to communicable disease control?
   1. Which are the most useful?
   2. What other journals or websites do you use frequently which are not on this list?
   3. Are there any journals on our list that should not be included on the list?
7. How would you define the concept of “evidence-based information?
   1. Do you use this concept in assessing the value of information you seek?

1. Do you feel there is a need to improve access to information related to communicable disease control?
   1. Is there a need to improve access specifically to evidence-based information?
   2. What are the biggest barriers or limitations you currently experience in trying to access the information you need?
2. What kinds of changes have you made in the way you disseminate information in response to suggestions from the end users you support.
3. How do you store information obtained that you plan to access again in the future?
