# Supplementary material for: Identifying strategies to improve access to credible and relevant information for public health professionals: a qualitative study
Source: BMC Public Health. 2006 Apr 5;6:89. doi: 10.1186/1471-2458-6-89 (PMC1456961; doi:10.1186/1471-2458-6-89)
Supplement: Additional File 2 — Script for CHP key informant interviews at DPH. [file 1471-2458-6-89-S2.doc]

# Script for CHP key informant interviews at DPH

# 8/11/2004

As part of a project funded by the CDC and ATPM (Association of Teachers of Preventive Medicine) and conducted by the UMass Medical School Library, we are conducting research to determine the external information access needs of public health professionals. In order to gain some preliminary focus for the project, we have already collected data from staff in the MDPH/ bureau of communicable disease control on their information accessing needs and challenges.

We are interested in the types of information from external sources that you need to do your work and the most effective information access mechanisms for professionals like yourselves. Your Director recommended that we talk to you along with a number of others in the Division of Community Health Promotion as those who most often need to access critical information related to your work. I would like to start by first asking you a few questions about your responsibilities related to information.

1. What is your job and how does it fit into the overall Division?
2. What kinds of questions come up in your work that you need to find answers for by accessing external information sources? (Give examples).
3. For the/one of the examples you just gave, what would be the steps in the process you would use to find the information to answer that question starting with the first thing you might do?
4. What role within your job responsibilities requires access to critical program-related information?
5. What kinds of program-related information do you need and what do you use each kind of information for? For example,
   1. Breaking news
   2. Emerging practice/programmatic information
   3. Well-established reference information
   4. Policies and guidelines
   5. Information that summarizes the evidence from all studies related to a particular question
   6. Other
6. Where do you currently get the program-related information you need?
   1. Web sites, web searches, online literature searches, online or hard copy journals, emails from co-workers, email distribution lists, online newsletters, books or other hard copy documents
   2. What information format is the most useful?
      1. original articles;
      2. summaries of original articles;
      3. critiques & commentaries or original research;
      4. systematic reviews, meta-analyses & evidence-based guidelines;
      5. comprehensive knowledge bases (one-stop shopping for Public Health information)
   3. Any other sources?

For offline information sources identified, continue with questions 13-16.

For online information sources identified, continue with questions 7-12.

1. What are your preferred methods for accessing this kind of information online?
2. What do you like most about this online information access methods?
3. What is the process you use to find the information you need online?
   1. Would you be willing to walk me through a recent example of this?
4. What would make this online information access method even more to your liking?
5. If you were able to access a credible summary or commentary of journal articles, would this be more helpful than direct access to full text of articles?
6. If you were able to receive a weekly email/listserve update specific to your area of interest that was pre-screened by experts, would you prefer this to being able to set up your own search criteria for weekly email information updates?

Go to question 17

1. What are your preferred methods for obtaining this kind of information?
2. What do you like most about obtaining information this way?
3. What is the process you use to find the information you need in this source?
   1. Would you be willing to walk me through a recent example of this?
4. What would make obtaining information you need from this source even more to your liking?

General

1. Considering all the sources of information and the information formats used in each, which sources and formats do you prefer, or are most useful to you?
2. How would you define the concept of “evidence-based information?
   1. Do you use this concept in assessing the value of information you seek?

1. Do you feel there is a need to improve access to information related to your program area?
   1. Is there a need to improve access specifically to evidence-based information?
   2. What are the biggest barriers or limitations you currently experience in trying to access the information you need?
2. How do you store information obtained that you plan to access again in the future?
