# Supplementary material for: Identifying strategies to improve access to credible and relevant information for public health professionals: a qualitative study
Source: BMC Public Health. 2006 Apr 5;6:89. doi: 10.1186/1471-2458-6-89 (PMC1456961; doi:10.1186/1471-2458-6-89)
Supplement: Additional File 3 — Some Focus Group questions for the DPH/CHP folks after the powerpoint presentation. [file 1471-2458-6-89-S3.doc]

**Some Focus Group questions for the DPH/CHP folks after the powerpoint presentation**

Introduce Power point PH Information Access Model as point of reference for questions below.

1. Is PubMed a good starting point for user-initiated searches or would you prefer something easier to use? What would make it easier?
2. Would you prefer more preformulated PH-specific searches of PubMed database such as those offered by Partners in Information Access for the Public Health Workforce? What topics would be of interest
3. Would you prefer to have searches run automatically for certain topics and results emailed to you periodically or do the searches yourself?
4. If you would like search results sent to you automatically, what topics would you have the most interest in and what keywords might you use to specify them them?
5. How important is an archiving feature such as the one provided by Safety Lit?

PubMed and Partners provide access to abstracts and to full text if you or your institution has a subscription. Journal Watch provides also commentaries on articles found in specific areas of interest. Commentaries generally highlight key findings and their significance for the general public.

1. Would commentaries on individual articles be helpful instead of or in addition to access to the abstracts and full text of articles?

Evidence-based Healthcare and Public Health is a journal that provides article summaries and critiques of methodology. They also have a website.

1. What is your level of interest in obtaining summaries of articles and critiques of their methodology?

National Guidelines Clearinghouse & Guide to Community Preventive Services provide access to systematic reviews and evidence-based guidelines

1. How much interest do you have in systematic reviews of collections of articles and evidence-based guidelines informed by these compared to the things we have discussed earlier?

UptoDate is a clinical example of a more encyclopedic information source with easy subtopic selection within topic areas

1. How helpful might it be to have access via a single website to more encyclopedic information about topics of interest that include not only current, regularly updated, synthesized, evidence-based information but also comprehensive background information and the points of view of experts for those areas where evidence is limited?
2. How useful would it be to have a sophisticated but easy-to-use indexing system that can allow you to access just the subset of information about the topic you are looking for?

Conferences seem to be a good source of information about emerging promising interventions.

1. How useful might it be to you to be able to search the topics that are being presented at recent relevant conferences and to obtain abstracts of these?
2. How useful might it be to also be able to search news media archives for topics of interest?

Re: model slide

1. If we were to develop a model such as the one on the slide with a rating system to screen new information for relevance and credibility, would you be willing to participate in a review/rating system for new information in your area of expertise?

Which one or two of the various features just mentioned or combination of these would meet your needs most effectively,
