# Supplementary material for: Identifying strategies to improve access to credible and relevant information for public health professionals: a qualitative study
Source: BMC Public Health. 2006 Apr 5;6:89. doi: 10.1186/1471-2458-6-89 (PMC1456961; doi:10.1186/1471-2458-6-89)
Supplement: Additional File 4 — Quotes Supporting Information Need Categories. Table of themes and supporting quotes. [file 1471-2458-6-89-S4.doc]

# Attached File 4: Quotes Supporting Information Need Categories

| **Feature** | **Informant Quotes** |
| --- | --- |
| **1. Early Reports on Health Risks** | “A really good example of something that would not appear in any clinical journals would be the HIV outbreak in the porn industry. This is a huge issue in STDs and is making headlines and I have to know about that, but it wouldn’t be picked up by these more traditional types of publications.”  “And it’s because we have to be so responsive, and so several times a day we get notification from EpiX about things that are happening. And then on the immunization side of things, particularly around smallpox preparedness, sometimes a couple of times a day or once a day, now it’s a couple times a week, we get updates about smallpox adverse events that are happening.” |
| **2. Emerging Practices** | “We also need promising practices, because I think evidence based interventions are limited.”  “We’re in listservs through CDC that is a general colleague kind of sharing of information, and of outbreaks. So it’s mainly information sharing that we’re getting of things that are happening in other parts of the country. So it’s not like sharing journal articles, it’s sharing specific incidents that are happening, so we can get more information that way of how people follow up, how do they do it – maybe we could do the same thing, and that sort of thing.”  “It is harder to get information regarding best and promising practices.” |
| **3. Evaluated Practices** | “So we’re now focusing more on identifying models that have been evaluated, that we can try to replicate in the state. So that kind of information is fairly important for us. Looking at what’s already been evaluated, even if they’re not “evidence based”, but that are promising models that may be useful to look at more closely.” |
| **4. Reference Information** | **“**I use them [i.e., online reference books] a lot, but the better ones were made for electronic searching. It’s the cross referencing and searching that is so valuable not just having it online.” |
| **5. Published Research** | “Generally article searches – yes. I’d have to say that overall CDC is someplace I would not turn to for that kind of information – for prevention, education and interventions. They don’t seem to have that data.”  “And now with the internet, I use the internet most of the time – I will do searches and get articles downloaded from there. “ |
| **6. Evidence-based Guidelines** | “Whatever we do has to be evidence-based. We have to make sure that whatever data that we come up with has to be accurate, and facts have to be checked out a couple of times before we insert it into our plan.”  “Absolutely. As a matter of fact, I think in terms of grant writing, pretty much they want you to be using evidence-based interventions, so that you really have to provide some background as to why you feel the intervention you are suggesting is evidence-based.” |
