# Supplementary material for: Identifying strategies to improve access to credible and relevant information for public health professionals: a qualitative study
Source: BMC Public Health. 2006 Apr 5;6:89. doi: 10.1186/1471-2458-6-89 (PMC1456961; doi:10.1186/1471-2458-6-89)
Supplement: Additional File 5 — Quotes Supporting Information Accessing Feature Needs and Preferences. Table of themes and supporting quotes. [file 1471-2458-6-89-S5.doc]

# Attached File 5: Quotes Supporting Information Accessing Feature Needs and Preferences

| **Feature** | **Informant Quotes** |
| --- | --- |
| **1. PH-specific keywords** | “A lot of government websites have a vast amount of information, but maybe because I do not understand the topic that I am looking for well enough, I don’t know the keywords to use … I want now to identify databases that a health economist should use to determine return on for worksites on disease management, demand management, health promotion [programs] and I have no idea what I am talking about so formulating a search around that [is difficult]. That’s why we are searching because we are trying to formulate what we should be doing.”  “A [keyword] category that would be common to a lot of our areas in PH would be ‘cost-effectiveness’. We usually have to make that argument for most of our policies. I’d have to think about it a little more, but I think there are some common categories. Another category for us would be ‘population-based’ studies.” |
| **2. Preformulated searches** | “Having done searches without a lot of criteria, you do end up with a really big list [of search keywords] and by doing it pre-formulated, you have already defined the question. I think that would be very, very useful. The challenge there is to define the questions. What is it you are looking for and not to narrow it so far that you miss out on the breadth of what is out there.”  “I think what you are saying is that ideally there would be a model on line where someone would go to find PH info where there would be an option to go to predefined searches for PH… It sort of depends what you are looking for, but anything that simplifies the search further would be helpful.” |
| **3. Automatic electronic notification** | “The other thing is just getting updates sent to you. We need to know what is going on. I ought to be able to get things on avian influenza and stuff like that. I would want to come up with a script and do a monthly search to see what’s new, what’s out there, what do I need to know about.”  “I am trying to look at the difference between the alert and the customized search facility. My email box gets very full and for me it would be more useful to go to the web and do it that way, but ideally I want to replicate what TB [Update] does. They do a very nice job out of CDC in terms of putting together the literature, but we don’t have that [kind of listserv] in our area, so I would go to the web to do it.” |

| **Feature** | **Informant Quotes** |
| --- | --- |
| **4. Abstracts, summaries, commentaries** | “If we have something nicely summarized, we can pass it on easily. But like in [some of] our areas, we work a lot with communities and populations where there is very little summarized. There is not a lot that comes out in the literature any way, but if there were any summaries, it would be very helpful.”  “They are helpful, yes. There aren’t a lot of them that I’ve noticed.”  “I like the critique better than the summary. Someone has really identified strengths and weaknesses and distilled it down.”  “Having a third party look at information is increasingly important.” |
| **5. Systematic Reviews** | “That is good. The more articles can be abstracted and annotated, that is extremely helpful. I would just want them over a broader area than what is available via the Community Guide – covering different disciplines.”  “It would be nice to go to one source, and have several things compared and studied, and some kind of an analysis done.” |
| **6. Evidence-based guidelines** | “Yes – [a database of evidence-based guidelines would be helpful]. But it would have to be simple, and summaries, and updated frequently.”  “Yes, we do [use] information about policies and guidelines [like the AHRQ Clearinghouse]. And guidelines from, say, the ADA (American Diabetes Association), or MHQP (Mass Health Quality Partnership), and so on.” |

| **Feature** | **Informant Quotes** |
| --- | --- |
| **7. Comprehensive Knowledge collection** | “That’s what I want access to [i.e., something like UpToDate.com].”  “It [PubMed] is a base, but other bases need to be added to it for other industries and professions. If you were trying to figure out what services insurance should cover and what is the return on that. Do you get that through actuary? I don’t even know what an actuary does. There is all that other side of health that is not contained in PubMed… I think the websites you shared were very helpful, the background info you shared. The broader base [of information] that we have to work with is key.”  “We are trying to reach out to non-PH groups that can help us with what we are trying to do in PH and PubMed wouldn’t necessarily do that, but I think it is a good start for what it offers. But I think we need access to other types of journals that are out there for research that has been conducted by other groups that we would be interested in… I absolutely think being able to pull information from a broader base is needed more than just clinical info.”  “I think there is also a dimension of experience and evidence associated with behavior modification and risk-factor reduction counseling that is important. Because not only are we interested in monitoring situations, but in changing behaviors through the programs so that is an additional dimension that PubMed might not bring.” |
| **8. Within Article Indexing** | “So much out there, and sometimes it takes us a long time to find the right source, the right web page, and then once we find the web page we have to read on many, many pages to get to what we want. And then we have to study it, and then do our own assessment of the information.”  “Indexing and organizing the information in a better way, for better access, then yes [there is a need for this]. Because the information we’ve been getting is good information.”  “I like it [within article indexing] myself. If I only wanted to know ‘recommended approaches’, it would be easier for me to find it” |
| 9. Archiving | “I use the TB [Update] quite a bit and I’ve taken to saving it. And I’ve always wondered if I’d ever go back to it. And sometimes I’ve tried to but I can never remember what week something came out. A piece of me wants to archive them but I don’t know how to find them again without being able to search by topic.”  “I really like that because you may only glance at [an article] at the time but you may need to go back to it in more detail at a later time, and I don’t want to keep it on my desk.” |
